# CT-based texture analysis predicts BRAF^V600E^ mutation in calcified papillary thyroid carcinoma

**DOI:** 10.3389/fonc.2025.1660725

**Published:** 2025-10-22

**Authors:** Yongqin Chen, Wenfu Cao, Hang Li, Shuxiang Chen, Liwan Zhang, Huijuan Zhang, Yongxiu Tong

**Affiliations:** ^1^ Department of Pathology, Fujian Medical University Union Hospital, Fuzhou, China; ^2^ Department of Radiology, Provincial Clinical Medical College of Fujian Medical University, Fujian Provincial Hospital,Fuzhou University Affiliated Provincial Hospital, Fuzhou, China; ^3^ Department of Radiology, Affiliated Hospital of Medical School, University of Electronic Science and Technology of China, Sichuan Academy of Medical Sciences and Sichuan Provincial People's Hospital, Chengdu, China; ^4^ Department of Health Checkup, Fujian Maternity and Child Health Hospital, Fuzhou, China

**Keywords:** papillary thyroid cancer, texture analysis, BRAF^V600E^, calcification, mutation

## Abstract

**Background:**

The BRAF gene plays an essential role in papillary thyroid carcinoma (PTC).

**Purpose:**

To investigate the potential of CT-based texture analysis in predicting BRAF^V600E^ mutation in calcified PTC.

**Material and methods:**

475 cases of calcified PTC from two centers, who underwent CT scans, surgery, and BRAF^V600E^ mutation testing, were included. Data from the first center were randomly divided into training and testing sets, whereas data from the second center constituted an external validation set. Using MaZda software, 256 texture features were extracted from both the parenchymal and calcified areas. The top ten texture feature parameters were selected by Fisher, minimization of both classification error probability and average correlation coefficients (POE+ACC), and mutual information measure (MI) feature selection algorithms. Data analysis and classification were performed using principal component analysis (PCA), linear discriminant analysis (LDA), and nonlinear discriminant analysis (NDA). Receiver operating characteristic curves were used to evaluate the diagnostic performance.

**Results:**

The NDA method demonstrated excellent diagnostic performance compared to the LDA and PCA methods, with error rates of less than 10%, less than 25%, and greater than 30%, respectively in the training and validation sets. For parenchymal and calcified areas of PTC, the POE+ACC+NDA and MI+NDA methods exhibited the lowest error rates, with an area under the curve (AUC) of 0.969 in the training set and 0.964 in the internal validation set. Conversely, the Fisher+PCA and MI+PCA methods had the highest error rates, with AUC values of 0.413 and 0.525 in the training set, and 0.433 and 0.560 in the internal validation set, respectively.

**Conclusion:**

The POE+ACC+NDA or MI+NDA method provided high diagnostic performance for predicting BRAF^V600E^ mutation in PTC. Texture analysis of tumor calcified area can also be used to predict BRAF^V600E^ mutation.

## Introduction

In recent decades, there has been a significant global rise in the incidence of thyroid cancer. The majority (over 80%) of cases belong to the papillary thyroid carcinoma (PTC) subtype (1). The 2015 American Thyroid Association Guidelines introduced active surveillance as a safe alternative to surgical intervention for low-risk PTCs. However, for high-risk PTCs, aggressive surgical treatment remains the only reliable and safe option (2). The BRAF^V600E^ gene mutation is closely associated with PTC and plays a crucial role in its pathogenesis. Previous study has demonstrated that PTC cases with the BRAF^V600E^ mutation exhibited a more aggressive behavior than those without such mutation (3). Therefore, accurately assess the BRAF^V600E^ mutation status is critically important for definitive treatment decisions.

Currently, the BRAF^V600E^ mutation tests are typically performed on preoperative invasive fine-needle aspiration biopsy samples or surgically resected specimens. However, the use of ultrasound-guided fine needle puncture is invasive and can lead to potential adverse effects, such as pain, bleeding, and acute thyroid swelling (4–6). Texture analysis, a well-established technique, is applied when performing quantitative analysis of tumor heterogeneity by analyzing the distribution and the relationship of pixels or voxel gray levels in the target region (7). The ease of obtaining texture information from routinely acquired images without additional imaging procedures is required, and accumulating data showing the correlation between heterogeneity and adverse tumor biology is a major advantage of the technique, which has been utilized to identify tumor differentiation degrees, assess tumor characteristics, and evaluate treatment efficacy (8, 9). While there have been limited studies on the use of texture analysis for predicting the molecular state of thyroid cancer, most of these studies have focused on ultrasound images (10, 11). Although ultrasound images exhibit high sensitivity and specificity in diagnosing thyroid cancers, there were some limitations such as operator and machine dependence, limited image data, and a lack of representative features. Conversely, CT imaging provides more intuitive and accurate information directly from CT scans. Additionally, CT imaging is particularly effective in visualizing calcification, especially large and marginal calcifications (12). However, when performing texture analysis, regions of interest (ROI) should be delineated while excluding calcifications, despite their presence in certain thyroid tumors and their significance in distinguishing between benign and malignant tumors (13). Further investigation is thus needed to determine whether the presence of calcifications influences the results of texture analysis.

The primary objective of this study is to investigate the potential of texture analysis base on the preoperative CT imaging, in predicting the presence of BRAF^V600E^ mutation in calcified PTC. Additionally, our study aims to compare the diagnostic performance of various texture feature extraction methods.

## Materials and methods

### Patients

**Figure 1:**
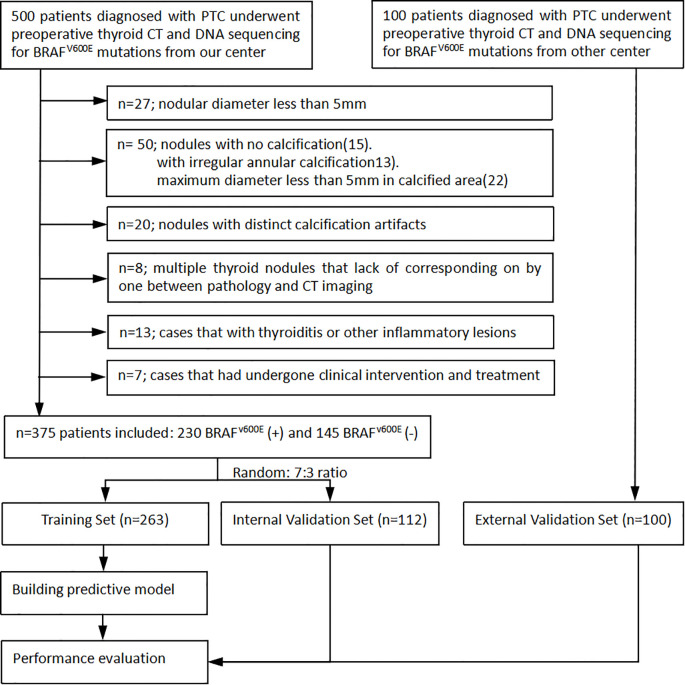
Flow diagram shows inclusion and exclusion criteria for the study.

We thoroughly reviewed the institutional databases from two centers to identify patients with surgically confirmed PTC with calcification. From January 2016 to December 2024, a total of 600 patients underwent preoperative thyroid CT scans and DNA sequencing to detect BRAF^V600E^ mutation. The following exclusion criteria were applied: 1) nodules with a diameter less than 5mm, as these are known to have low accuracy using the ROI method (14); 2) nodules without calcification or with irregular annular calcification, or with a maximum diameter less than 5mm in the calcified area to avoid volume effect; 3) nodules with obvious calcification artifacts that hindered observation and the delineation of ROI; 4) cases with multiple thyroid nodules lack of precise correlation between pathology and CT imaging; 5) cases with thyroiditis or other inflammatory lesions; and 6) cases that had undergone prior clinical interventions. Finally, the remaining 475 patients were included in this study (**Figure 1**). A primary cohort of 375 patients from the first center was randomly divided into a training set and an internal validation set in a 7:3 ratio, while an additional 100 patients from the second center constituted the external validation set. This study was approved by the institutional review board of Fujian Provincial Hospital (approve number: K2021-12-029), and the requirement for obtaining written informed consent was waived due to the retrospective nature of the study.

### CT examinations

All the patients included in the study underwent unenhanced and venous phase contrast-enhanced 64-slice spiral CT scans. The scanning region encompassed from the pharynx to the upper edge of the clavicle. In cases of posterior sternal thyroid involvement, the scanning range was extended to the level of tracheal bifurcation. The acquisition parameters were set as follows: tube voltage of 120kVp, tube current of 250mA, detector collimation of 0.625mm×64, pitch of 0.938, rotation time of 0.5s, and slice thickness of 3.75mm. For contrast-enhanced scanning, 75mL of iodinated contrast agent was intravenously injected through the ulnar vein at a flow rate of 3.5mL/s. The scan delay for the venous phases was set at 45-50s.

### Texture feature analysis

**Figure 2:**
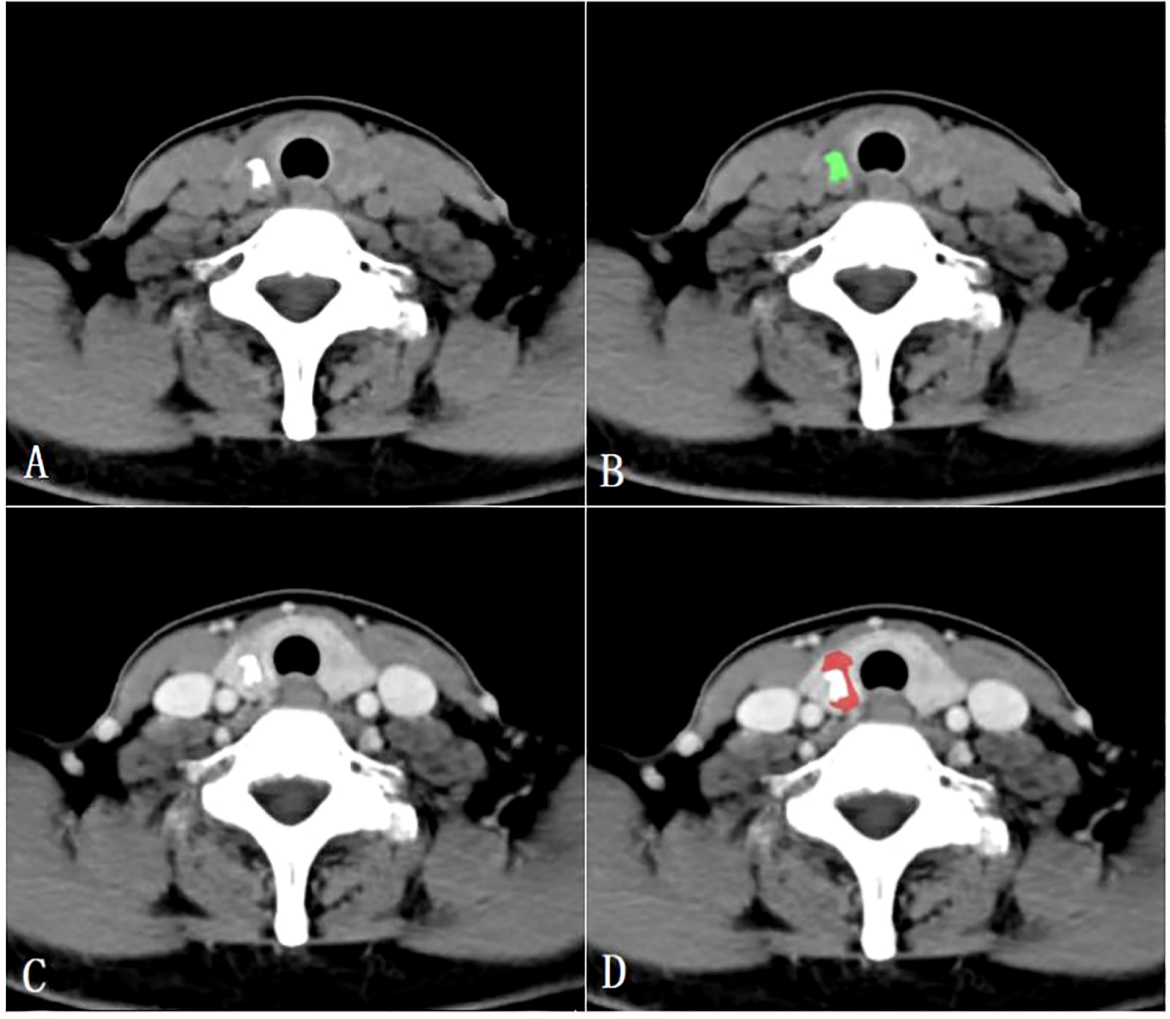
Region of interest placement in the calcified area of the papillary thyroid carcinoma on unenhanced CT images **(A, B)**; Region of interest placement in the parenchymal area of the papillary thyroid carcinoma on venous phase CT images **(C, D)**.

All CT images were retrospectively analyzed and evaluated using the picture archiving and communication systems (PACS) by two radiologists with 15 years (radiologist #1) and 8 years (radiologist #2) of expertise in diagnosing thyroid disease. Subsequently, the images were imported into the MaZda software (version 4.6, Instytut Elektroniki, Technical University of Lodz, Poland) (15) for further analysis. The two radiologists reached a consensus and manually outlined the margins of the parenchymal area on the maximum central level of the tumor, as well as the calcified area on the maximum calcified level of the tumor to define the ROIs. To ensure accurate delineation and to minimize the influence of the interference of CT contrast agents on calcification and calcified artifacts in the parenchymal area, the ROIs for the calcified areas were outlined on unenhanced CT scan images, while the ROIs for the parenchymal areas were outlined on enhanced venous phase CT images (**Figure 2**).

Textural analysis (TA) was performed using the MaZda software in three sequential steps: 1) Extraction of texture feature parameters: Mazda offered six texture analysis methods, such as gray histogram and absolute gray gradient. From each ROI, a total of 256 texture feature parameters were derived using the combination of these six analysis methods. 2) Selection of relevant features: Feature selection algorithms included the Fisher coefficient, probability of classification error combined with average correlation coefficients (POE+ACC), and mutual information measure (MI). Each algorithm yielded 10 optimal texture feature parameters. 3) Dimensionality reduction and feature classification: Three dimensionality reduction methods available in the B11 programs, namely principal component analysis (PCA), linear discriminant analysis (LDA), and nonlinear discriminant analysis (NDA), were used to reduce dimensionality. Subsequently, the software classified the lesions based on the aforementioned results and computed the error rate (R) for predicting BRAF^V600E^ mutation (R = the number of incorrectly classified lesions/the total number of lesions). The results of texture analysis were categorized into five grades according to R values: excellent (0–10%), good (11%–20%), moderate (21%–30%), fair (31%–40%), and poor (41%–100%).

To address the class imbalance between BRAF^V600E^ mutation-positive (n=162) and negative (n=101) cases in the training set, a weighted cross-entropy loss function was employed during model training. The weights were set to be inversely proportional to class frequencies, thereby increasing the penalty for misclassifying minority class (negative) samples.

### BRAF^V600E^ mutation analysis

The BRAF^V600E^ mutation analysis in PTC surgical specimens was performed at the Department of Pathology at our hospital. Based on the findings, all cases were categorized as either positive or negative for the BRAF^V600E^ mutation.

### Statistical analysis

Statistical analysis was performed using SPSS 23.0, MedCalc 19.8, and R 4.0.3. To compare the BRAF^V600E^ mutation–positive and –negative cases of PTCs, we used Chi-square or Fisher exact tests for categorical variables. Receiver operating characteristic (ROC) curve analysis was performed, and the area under the curve (AUC), 95% Confidence Interval (CI), sensitivity, and specificity were calculated to evaluate the diagnostic performance of the combined parameters. The optimal model underwent comprehensive evaluation using ROC curves, calibration curves, and decision curve analysis (DCA). Delong’s test was utilized to evaluate the differences in diagnostic performance for the BRAF^V600E^ mutation between the parenchymal and calcified tumor regions across the training, internal validation and external validation sets. A *P* value < 0.05 was considered statistically significant.

## Results

### General characteristics of the patients

The training set consisted of 263 cases (192 females, 71 males), with a mean age of 41.1 ± 13.3 years. The BRAF^V600E^ mutation was present in 162 cases (61.6%) and absent in 101 cases (38.4%). The internal validation set included 112 cases (83 females, 29 males) and had a mean age of 42.5 ± 11.7 years. Within this set, 68 cases (60.7%) were BRAF^V600E^ -positive and 44 cases (39.3%) were BRAF^V600E^ -negative. No significant differences existed between the training and internal validation sets in age, gender, or BRAF^V600E^ mutation (*P* = 0.173, *P* = 0.312, and *P* = 0.448, respectively). The external validation set consisted of 100 cases (77 females, 23 males) with a mean age of 46.3 ± 8.28 years. Among these, 50 cases were BRAF^V600E^-positive and 50 cases were BRAF^V600E^-negative. Significant differences in age, gender, and BRAF^V600E^ mutation were observed between the external validation and training sets(all P<0.001) (**Table 1**).

**Table 1:** The general characteristics of the patients in training and validation sets.

Items	Training set (n=263)	Internal validation set (n=112)	External validation set (n=100)	P value ^1^	P value ^2^
Sex				0.31	<0.001
M	71 (27.0%)	29 (25.9%)	23 (23%)		
F	192 (73.0%)	83 (74.1%)	77 (77%)		
Age (mean) (yr)	41.1 ± 13.3	42.5 ± 11.7	46.3 ± 8.28	0.17	<0.001
BRAF^V600E^ Mutation				0.45	<0.001
BRAF^V600E^ (+)	162 (61.6%)	68 (60.7%)	50 (50%)		
BRAF^V600E^ (-)	101 (38.4%)	44 (39.3%)	50 (50%)		

P Value ^1^, comparison result between the training set and internal validation set; P Value ^2^, comparison result between the training set and external validation set.

### Texture analysis

**Table 2** presents the BRAF^V600E^ mutation (R) results derived from various texture analysis methods applied to the parenchymal and calcified areas of PTCs in training, internal validation and external validation sets. The NDA method, applied to either the parenchymal or calcified areas of PTCs, along with any of the feature selection algorithms, demonstrated excellent diagnostic performance in predicting the BRAF^V600E^ mutation, with an R value of less than 10% across the training, internal validation and external validation sets. The LDA method exhibited good diagnostic performance, with an R value of less than 25% in all three sets. On the other hand, the PCA method showed poorer diagnostic performance, with the highest R value exceeding 30% in each set.

**Table 2:** The error rate of BRAF^V600E^ mutation for the different dimensionality reduction method in the parenchymal and calcified area of PTCs.

Tumor area	Dimensionality reduction method		Fisher	POE+ACC	MI
Parenchymal area	PCA	Training Set	53.85	46.15	34.62
		Internal Validation Set	52.17	43.48	34.78
		External Validation Set	56.10	46.34	36.58
	LDA	Training Set	7.69	19.23	7.69
		Internal Validation Set	8.70	21.74	8.70
		External Validation Set	9.76	19.51	9.76
	NDA	Training Set	7.69	3.85	7.69
		Internal Validation Set	8.70	4.35	8.70
		External Validation Set	9.76	4.88	9.76
Calcified area	PCA	Training Set	38.46	34.62	42.31
		Internal Validation Set	34.78	34.78	39.13
		External Validation Set	40.00	36.67	43.33
	LDA	Training Set	11.54	7.69	23.08
		Internal Validation Set	13.04	8.70	26.08
		External Validation Set	16.67	13.33	26.67
	NDA	Training Set	7.69	7.69	3.85
		Internal Validation Set	8.70	8.70	4.35
		External Validation Set	10.00	10.00	6.67

PCA, principal component analysis; LDA, linear discriminant analysis; NDA, nonlinear discriminant analysis; POE+ACC, minimization of both classification error probability and average correlation coefficients; MI, Mutual information.

Regarding the parenchymal area of PTCs, the combination of POE+ACC with the NDA method yielded the lowest R values of 3.85% in the training set and 4.35% in the internal validation set for predicting BRAF^V600E^ mutation. Conversely, the Fisher with PCA method combination provided the highest R values of 53.85% in the training set and 52.17% in the internal validation set for predicting the mutation. Consistent results were obtained in the external validation set, where the lowest and highest R values were 4.88 % and 56.10%, respectively.

As for the calcified area of PTCs, the combination of MI with the NDA method provided the lowest R values of 3.85% in the training set and 4.35% in the internal validation set for predicting BRAF^V600E^ mutation. Conversely, the combination of MI with the PCA method yielded the highest R values of 42.31% in the training set and 39.13% in the internal validation set. Similarly, the external validation set demonstrated consistent findings, with the lowest and highest R values being 6.67 % and 43.33%, respectively.

### Efficiency of the predictive model

**Figure 3:**
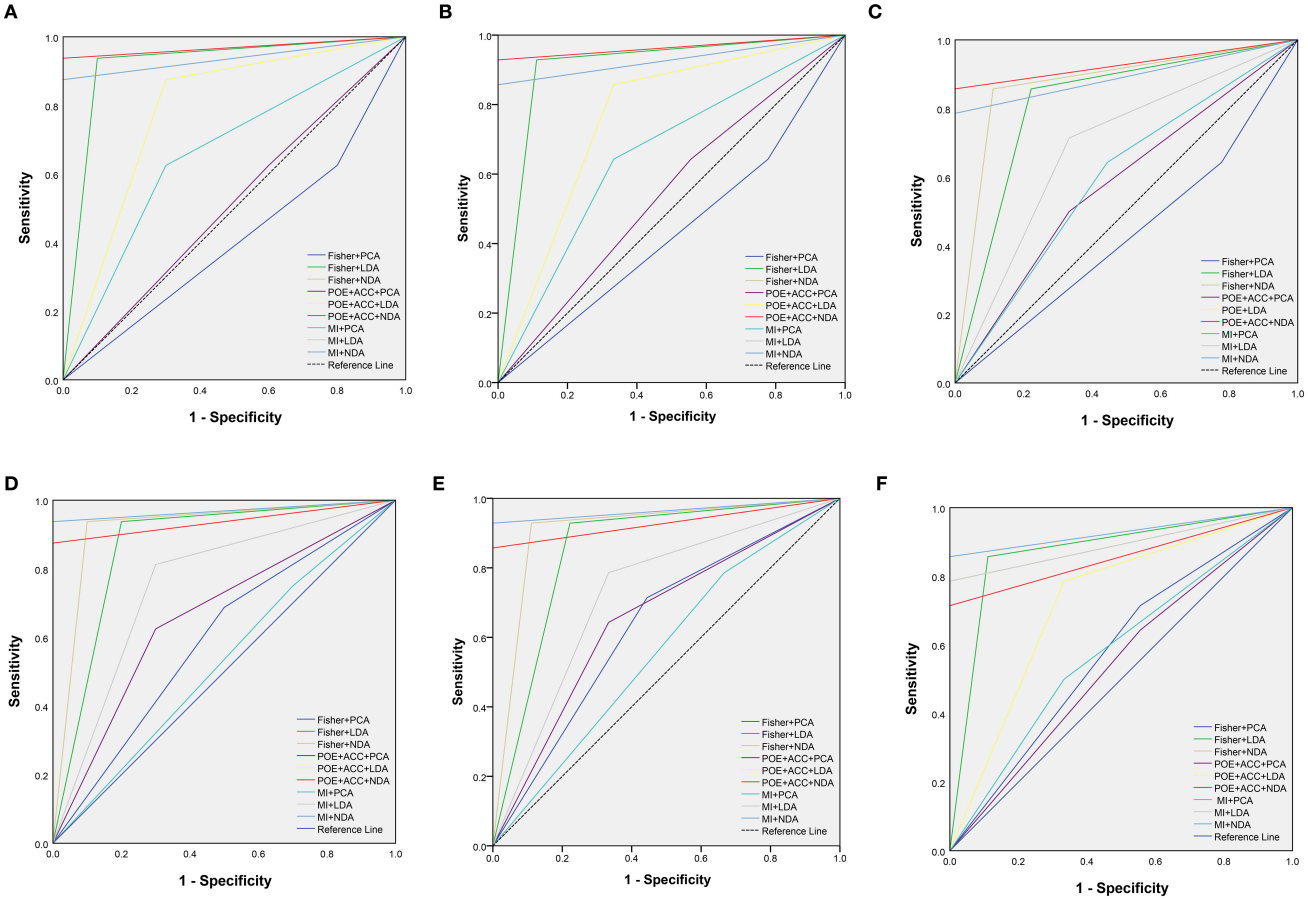
The receiver operating characteristic curves of combined different texture analysis methods in the parenchymal area **(A–C)** and calcified area **(D–F)** of papillary thyroid carcinoma for predicting BRAF^V600E^ mutation in the training, internal validation and external validation sets.

ROC curve were utilized to evaluate the diagnostic efficacy of various combined texture analysis methods in predicting the BRAF^V600E^ mutation in PTCs within the training, internal validation and external validation sets. The results are presented in **Tables 3**, **4** and **Figure 3**. Across the training and validation sets, the AUC of PCA methods ranged from 0.5 to 0.7 for both the parenchymal and calcified areas of PTCs. The AUC of LDA methods ranged from 0.7 to 0.94, while the AUC of NDA methods exceeded 0.9. Notably, the combination of POE+ACC with the NDA method for the parenchymal area and the combination of MI with the NDA method for the calcification area exhibited the highest AUC values of 0.969 (95%, 0.908-1.000) in the training set and 0.964 (95%, 0.894-0.999) in the internal validation set. Conversely, the combination of Fisher with PCA method based on parenchymal area and the combination of MI with PCA method based on calcification area demonstrated the lowest AUC values of 0.413 (95%, 0.408-0.767) and 0.525 (95%, 0.493-0.671) in the training set, and 0.433 (95%, 0.392-0.474) and 0.560 (95%, 0.482-0.630) in the internal validation set, respectively. Consistent results were obtained in the external validation cohort, where the highest AUC values for both the parenchymal and calcified areas were 0.929 (95%, 0.858-0.996), and the lowest AUC values were 0.433 (95%, 0.431-0.562) and 0.544 (95%, 0.497-0.629), respectively. Importantly, no significant disparity in diagnostic performance for predicting the BRAF^V600E^ mutation was observed between the parenchymal and calcified areas of the tumor across the training, internal validation and external validation sets (**Table 5**).

**Table 3:** Evaluation of diagnostic performance of BRAF^V600E^ mutation in parenchymal area of PTCs by combined different texture analysis methods.

Feature selection algorithm	Dimensionality reduction method	Training set				Internal validation set				External validation set			
		Sensitivity (%)	Specificity (%)	AUC	95%CI	Sensitivity (%)	Specificity (%)	AUC	95%CI	Sensitivity (%)	Specificity (%)	AUC	95%CI
Fisher	PCA	20.0	62.5	0.413	0.408-0.767	22.2	64.3	0.433	0.392-0.474	64.3	22.2	0.433	0.431-0.562
	LDA	90.0	93.7	0.919	0.803-0.927	88.9	92.9	0.909	0.878-0.932	85.7	77.8	0.817	0.627-0.999
	NDA	100	87.5	0.938	0.854-0.943	100	85.7	0.929	0.904-0.969	85.7	88.9	0.873	0.710-0.999
POE+ACC	PCA	40.0	62.5	0.513	0.511-0.714	44.4	64.3	0.544	0.513-0.611	50.0	66.7	0.583	0.508-0.641
	LDA	70.0	87.5	0.788	0.616-0.795	66.7	85.7	0.762	0.720-0.819	78.6	100	0.893	0.855-0.927
	NDA	100	93.7	0.969	0.908-1.000	100	92.9	0.964	0.894-0.999	85.7	100	0.929	0.858-0.996
MI	PCA	70.0	62.5	0.663	0.569-0.856	66.7	64.3	0.655	0.597-0.686	64.3	55.6	0.599	0.561-0.642
	LDA	100	87.5	0.938	0.854-0.951	100	85.7	0.929	0.814-0.930	71.4	66.7	0.690	0.561-0.720
	NDA	100	87.5	0.938	0.854-0.960	100	85.7	0.929	0.873-0.951	78.6	100	0.893	0.855-0.975

PCA, principal component analysis; LDA, linear discriminant analysis; NDA, nonlinear discriminant analysis; POE+ACC, minimization of both classification error probability and average correlation coefficients; MI, mutual information; AUC, area under curve.

**Table 4:** Evaluation of diagnostic performance of BRAF^V600E^ mutation in calcified area of PTCs by combined different texture analysis methods.

Feature selection algorithm	Dimensionality reduction method	Training set				Internal validation set				External validation set			
		Sensitivity (%)	Specificity (%)	AUC	95%CI	Sensitivity (%)	Specificity (%)	AUC	95%CI	Sensitivity (%)	Specificity	AUC	95%CI
Fisher	PCA	50.0	68.8	0.594	0.533-0.619	55.6	71.4	0.635	0.587-0.701	71.4	55.6	0.579	0.529-0.625
	LDA	80.0	93.8	0.869	0.724-0.910	77.8	92.9	0.853	0.756-0.890	85.7	88.9	0.873	0.830-0.910
	NDA	90.0	93.8	0.919	0.803-0.931	88.9	92.9	0.909	0.853-0.929	78.6	100	0.893	0.800-0.955
POE+ACC	PCA	70.0	62.5	0.663	0.649-0.752	66.7	64.3	0.655	0.610-0.732	64.3	44.4	0.583	0.508-0.641
	LDA	100	87.5	0.938	0.854-0.959	100	85.7	0.929	0.899-0.992	78.6	66.7	0.726	0.673-0.773
	NDA	100	87.5	0.938	0.854-0.947	100	85.7	0.929	0.885-0.973	71.4	100	0.857	0.700-0.870
MI	PCA	30.0	75.0	0.525	0.493-0.671	33.3	78.6	0.560	0.482-0.630	50.0	66.7	0.544	0.497-0.629
	LDA	70.0	81.2	0.756	0.577-0.763	66.7	78.6	0.726	0.639-0.796	78.6	100	0.893	0.755-0.902
	NDA	100	93.8	0.969	0.908-1.000	100	92.9	0.964	0.894-0.999	64.3	44.4	0.929	0.858-0.996

PCA, principal component analysis; LDA, linear discriminant analysis; NDA, nonlinear discriminant analysis; POE+ACC, minimization of both classification error probability and average correlation coefficients; MI, mutual information; AUC, area under curve.

**Table 5:** Delong test for the combination with highest and lowest AUC between parenchymal and calcified areas in training and validation tests.

Combination	Group	Parenchymal area	Calcified area	P value
With highest AUC	Training Set	POE+ACC+NDA	MI+NDA	1.000
	Internal Validation Set	POE+ACC+NDA	MI+NDA	1.000
	External Validation Set	POE_ACC+NDA	MI+NDA	0.166
With lowest AUC	Training Set	Fisher+PCA	MI+PCA	0.719
	Internal Validation Set	Fisher+PCA	MI+PCA	0.966
	External Validation Set	Fisher+PCA	MI+PCA	0.464

	Tumor area	Group		
With highest AUC	Parenchymal area	Training Set vs. Internal Validation Set		0.317
		Training Set vs. External Validation Set		0.898
		Internal Validation Set vs. External Validation Set		0.741
	Calcified area	Training Set vs. Internal Validation Set		0.085
		Training Set vs. External Validation Set		0.822
		Internal Validation Set vs. External Validation Set		0.967
With lowest AUC	Parenchymal area	Training Set vs. Internal Validation Set		0.317
		Training Set vs. External Validation Set		0.569
		Internal Validation Set vs. External Validation Set		1.000
	Calcified area	Training Set vs. Internal Validation Set		0.966
		Training Set vs. External Validation		0.712
		Internal Validation Set vs. External Validation Set		1.000

**Figure 4:**
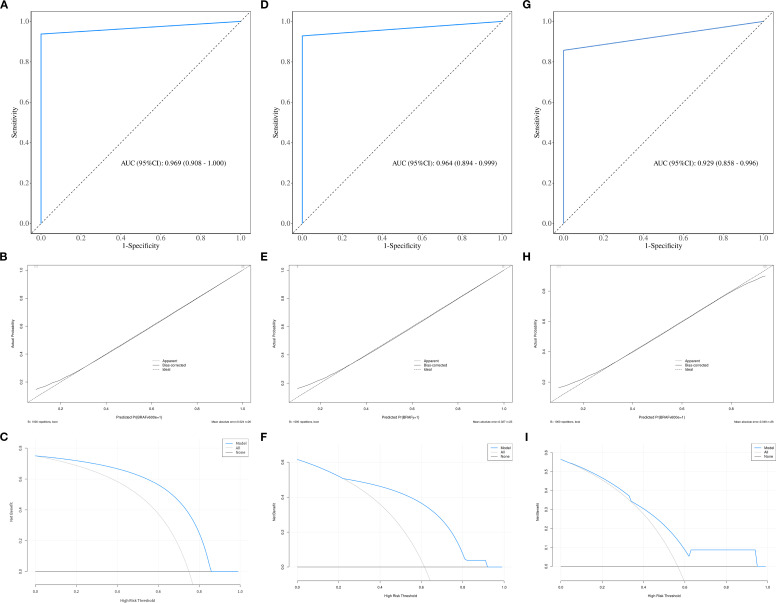
The ROC, calibration, and decision curves of the optimal prediction model in parenchymal area of PTCs across the training **(A–C)**, internal validation **(D–F)** and external validation sets **(G–I)**.

**Figure 5:**
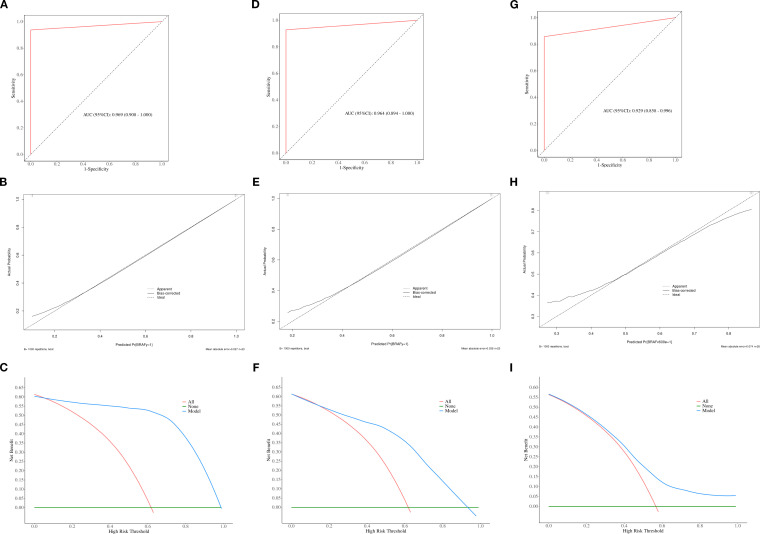
The ROC, calibration, and decision curves of the optimal prediction model in calcified area of PTCs across the training **(A–C)**, internal validation **(D–F)** and external validation sets **(G–I)**.

The calibration curve and DCA further assessed the optimal prediction models for both the parenchymal and calcified areas of PTCs in the training set, as illustrated in **Figures 4**, **5**. The calibration curve closely followed the ideal curve, which indicates high calibration accuracy and confirms the model’s strong potential for predicting BRAF gene mutation in papillary thyroid carcinoma (PTC). According to the DCA results, the model offered a clinical net benefit across a wide threshold probability range of 10% to 90%, surpassing both the “treat-all” and “treat-none” strategies.

Furthermore, to evaluate the robustness of the model’s conclusions, a sensitivity analysis was conducted. The performance of the model trained with class weights was compared with that of the model trained using the Synthetic Minority Over-sampling Technique (SMOTE) on an independent test set. The results revealed no significant difference in the area under the receiver operating characteristic curve (AUC-ROC) between the two models (0.924 vs. 0.916, respectively), while the SMOTE-trained model exhibited a slight improvement in recall for negative cases. These findings indicate that that our primary results are not substantially influenced by class imbalance.

## Discussion

Our study revealed that the utilization of MaZda software for texture analysis of preoperative CT images of PTCs enables the prediction of the BRAF^V600E^ mutation. Notably, when considering either the parenchymal or calcified areas of the tumor, the NDA method exhibited excellent diagnostic performance in conjunction with any of the feature selection algorithms. Specifically, the combination of the POE+ACC algorithm with the NDA method, as well as the combination of the MI algorithm with the NDA method, demonstrated the highest diagnostic performance in predicting the BRAF^V600E^ mutation in PTC. Furthermore, we meticulously delineated ROIs in both the parenchymal and the calcified areas of the tumor. Remarkably, our findings indicated that there was no significant difference in diagnostic performance between these two distinct areas.

PTC patients with BRAF^V600E^ positivity exhibited more aggressive clinical behavior, as determined by the constitutive abnormal activation of the mitogen-activated protein kinase (MAPK) signaling pathway driven by the BRAF^V600E^ mutation, along with its downstream cascade of complex molecular and cellular biological effects (16, 17). In addition, PTC displays significant gender disparity. Epidemiological studies have demonstrated that its occurrence rate in females is approximately 3–4 times higher than in males, which aligns with our study’s female-to-male ratio of approximately 3:1 (18). This phenomenon may be attributed to sex hormone (particularly estrogen)-mediated activation of oncogenic pathways, X chromosome-driven establishment of a gender-specific tumor microenvironment, and differences in healthcare-seeking behaviors between sexes (18–20).

In this study, the freely available software MaZda was use for feature extraction and dimensionality reduction. MaZda provides several feature extraction methods, including Fisher, POE+ACC, and MI, along with dimensionality reduction techniques such as PCA, LDA, and NDA (21, 22). PCA evaluates the covariance matrix of data, while LDA calculates the scattering matrix. Both methods use linear transformation for data linear classification. However, although PCA can effectively represent the dataset, its performance in data classification is relatively subpar (23). Our study supports these findings, as we found that the PCA method yielded unreliable results. Similarly, while LDA demonstrated accuracy in certain data classifications, it imposed high requirements on data characteristics, resulting in overall suboptimal diagnostic performance. Conversely, most datasets in our study consisted of non-linear data, which can be better classified using non-linear classifiers such as NDA instead of linear classifiers. NDA employs neural networks to perform non-linear transformations on data, projecting them into lower-dimensional feature spaces for linear classification (24, 25). Given the tumor heterogeneity, partial tumor cells serve as the basis for BRAF^V600E^ mutation. Additionally, BRAF^V600E^ mutation is associated with various factors, including tumor size, multifocality, tumor stage, aggressiveness, and the presence of Hashimoto’s thyroiditis (26, 27). Consequently, we can conclude that NAD is more suitable than the other two methods for predicting BRAF^V600E^ mutation, as it achieves higher accuracy by effectively modeling the non-linear characteristics of the dataset collected from BRAF^V600E^ mutations. Our study demonstrated that NDA exhibited excellent diagnostic performance, with an error rate of less than 10% in both the parenchymal and calcified areas, irrespective of the feature selection algorithm utilized.

Previous research has demonstrated that the MaZda software holds promise in distinguishing between benign and malignant tumors. For instance, Ardakani et al. (28) revealed that a combination of POE+ACC with NDA yielded excellent diagnostic performance in differentiating thyroid nodules using ultrasound images, achieving an accuracy of 97.14%, which closely aligns with our findings. However, their study only employed the Fisher and POE +ACC algorithms. On the other hand, our study revealed that the combination of MI with NDA also exhibited excellent diagnostic performance, with an AUC of 0.969. In contrast, Kwon et al. (10) observed that various dimensionality reduction and separator techniques had minimal influence on the results, while the choice of feature selection algorithm significantly impacted performance. Consequently, they concluded that radiomics studies using thyroid sonography have limited predictive ability for the BRAF mutation status of PTC. Yoon et al. (11) reported similar findings. Our study demonstrated that the NDA method achieved high accuracy, with an AUC exceeding 0.9. We hypothesize that the utilization of different radiomics software may have contributed to the discrepancies, highlighting one of the major challenges in current radiomics research. Furthermore, due to the diversity of texture feature analysis methods, the results can vary depending on the chosen methods, even within the same software. Therefore, radiomics research must establish unified and standardized protocols.

The majority of previous radiomics studies have emphasized the avoidance of calcification when delineating ROIs. However, it is worth noting that calcification not only is prevalent in thyroid tumors but also serves as a significant differential diagnostic indicator. While ultrasound plays a crucial role in thyroid tumor diagnosis, the elimination of calcification when delineating ROI, particularly in cases of microcalcification, poses challenges. Currently, whether calcification in thyroid tumors should be excluded during ROI delineation remains uncertain. For example, some previous studies have included calcification when drawing the ROIs and have discovered that ultrasound and MRI radiomics can be used to evaluate thyroid nodules (28–30). Conversely, other studies have suggested that calcification should be excluded (31, 32). In our study, we delineated the ROI separately in the parenchymal and calcified areas of the tumor. We confirmed that drawing the ROI in the calcified area can be also used to predict the BRAF^V600E^ mutation. There was no significant difference in diagnostic performance between these two areas. Two potential explanations may account for this finding. Firstly, the neovascularization and fibrous tissue in thyroid tumors are prone to cause calcium salt deposition due to the rapid proliferation of cancer cells. Additionally, the tumor itself secretes glycoprotein and mucopolysaccharide, which may contribute to nodule calcification. Secondly, microcalcification can be observed in 50% PTC cases and has been confirmed as an important factor associated with the BRAF^V600E^ mutation in PTC (26, 33).

This study had several limitations. Firstly, the data were processed using the default format of the Mazda software. Whether this affects the results requires further investigation. Secondly, we only focused on nodules larger than 5 mm and excluded those with thyroiditis or other inflammatory lesions. Furthermore, the mutation analysis was performed in a specific subgroup of PTCs. Therefore, our study did not encompass the mutation features of other types of thyroid cancer. Thirdly, texture analysis was performed on venous-phase images, as tumor tissues exhibit clearer boundaries and more stable texture features during this phase. Considering the potential influence of varying delay times on texture features and their diagnostic performance, a standardized delay of 45–50 seconds was employed for venous-phase imaging in this study. This aspect warrants further investigation. Finally, the retrospective design introduced variability in CT scanning parameters, which may have affected feature extraction and introduced measurement bias. Incomplete clinical data, such as thyroid function laboratory indicators, also somewhat limited the generalizability of the results. Future investigations will address these factors through prospective, multicenter studies to validate the present findings.

## Conclusion

In conclusion, our findings demonstrated that texture analysis based on preoperative CT images of PTCs using MaZda software can predict the presence of the BRAF^V600E^ mutation. The combination of POE+ACC with the NDA method or MI with the NDA method exhibited the highest diagnostic performance in predicting the BRAF^V600E^ mutation in PTC. The calcified area of the tumor can be utilized for predicting the BRAF^V600E^ mutation, and there was no significant difference in diagnostic performance between the parenchymal and calcified areas. Nevertheless, our results should be further validated in a larger sample size to enhance their potential clinical utility.

## Data Availability

The original contributions presented in the study are included in the article/supplementary material. Further inquiries can be directed to the corresponding author.

## References

[1] Hong X Li J Duan S Yon Y. Retrospective study of BRAFV600E mutation and CT features of papillary thyroid carcinoma. PeerJ. (2024) 12:38282867. doi: 10.7717/peerj.16810, PMID: 38282867 PMC10821721

[2] Haugen BR Alexander EK Bible KC Doherty GM Mandel SJ Nikiforov YE. 2015 American thyroid association management guidelines for adult patients with thyroid nodules and differentiated thyroid cancer: the American thyroid association guidelines task force on thyroid nodules and differentiated thyroid cancer. Thyroid. (2016) 26:1–133. doi: 10.1089/thy.2015.0020, PMID: 26462967 PMC4739132

[3] Hahn SY Kim TH Ki CS Kim SW Ahn S Shin JH. Ultrasound and clinicopathological features of papillary thyroid carcinomas with BRAF and TERT promoter mutations. Oncotarget. (2017) 8:108946–57. doi: 10.18632/oncotarget.22430, PMID: 29312581 PMC5752494

[4] Liu T Tilak M Awad S Lakoff J. A literature review of factors associated with pain from fine needle aspiration biopsy of thyroid nodules. Endocr Pract. (2022) 28:628–36. doi: 10.1016/j.eprac.2022.03.007, PMID: 35306164

[5] Imaoka K Nishihara M Nambu J Yamaguchi M Kawasaki Y Sugino K. Acute diffuse thyroid swelling after fine-needle aspiration: a case report and review of the literature. J Clin Ultrasound. (2021) 49:720–3. doi: 10.1002/jcu.23008, PMID: 33908030

[6] Liao LJ Lo WC Hsu WL Cheng PW Wang CP. Assessment of pain score and specimen adequacy for ultrasound-guided fine-needle aspiration biopsy of thyroid nodules. J Of Pain Res. (2017) 66:61–6. doi: 10.2147/JPR.S148088, PMID: 29343981 PMC5749542

[7] Chen S Zhang H Wei H Tong Y Chen X. Practical nomogram based on comprehensive CT texture analysis to preoperatively predict peritoneal occult metastasis of gastric cancer patients. Front Oncol. (2022) 12:1–12. doi: 10.3389/fonc.2022.882584, PMID: 36531010 PMC9753569

[8] Yu J Deng Y Liu T Zhou J Jia X Xiao T. Lymph node metastasis prediction of papillary thyroid carcinoma based on transfer learning radiomics. Nat Commun. (2020) 11:4807–7. doi: 10.1038/s41467-020-18497-3, PMID: 32968067 PMC7511309

[9] Colakoglu B Alis D Yergin M. Diagnostic value of machine learning-based quantitative texture analysis in differentiating benign and Malignant thyroid nodules. J Oncol. (2019) 2019:6328329. doi: 10.1155/2019/6328329, PMID: 31781216 PMC6874925

[10] Kwon MR Shin JH Park H Cho H Hahn SY Park KW. BRAF radiomics study of thyroid ultrasound for predicting mutation in papillary thyroid carcinoma: preliminary results. AJNR Am J Neuroradiol. (2020) 41:700–5. doi: 10.3174/ajnr.A6505, PMID: 32273326 PMC7144636

[11] Yoon JH Han K Lee E Lee J Kim EK Moon HJ. Radiomics in predicting mutation status for thyroid cancer: a preliminary study using radiomics features for predicting BRAFV600E mutations in papillary thyroid carcinoma. PLoS One. (2020) 15:e0228968. doi: 10.1371/journal.pone.0228968, PMID: 32053670 PMC7018006

[12] Wu JH Zeng W Wu RG Wang M Ye F Fu MY. Comparison of ultrasonography and CT for determining the preoperative benign or Malignant nature of thyroid nodules: diagnostic performance according to calcification. Technol Cancer Res Treat. (2020) 19:1–6. doi: 10.1177/1533033820948183, PMID: 32940552 PMC7506781

[13] Nabahati M Ghaemian N Moazezi Z Mehraeen R. Different sonographic features of peripheral thyroid nodule calcification and risk of Malignancy: a prospective observational study. Pol J Radiol. (2021) 86:e366–71. doi: 10.5114/pjr.2021.107450, PMID: 34322186 PMC8297479

[14] Tessler FN Middleton WD Edward G Grant EG. ACR thyroid imaging, reporting and data system (TI-RADS): white paper of the ACR TIRADS committee. J Am Coll Radiol. (2017) 14:587–95. doi: 10.1016/j.jacr.2017.01.046, PMID: 28372962

[15] Strzelecki M Szczypinski P Materka A. MaZda 4.00 MR Analysis Software: User’s Manual. Available online at: https://qmazda.p.lodz.pl/ (Accessed September 20, 2025).

[16] Tsou P Wu CJ. Classifying driver mutations of papillary thyroid carcinoma on whole slide image: an automated workflow applying deep convolutional neural network. Front Endocrinol (Lausanne). (2024) 15:1395979. doi: 10.3389/fendo.2024.1395979, PMID: 39564124 PMC11573888

[17] Zhu G Deng Y Pan L Ouyang W Feng H Wu J. Clinical significance of the BRAFV600E mutation in PTC and its effect on radioiodine therapy. Endocr Connect. (2019) 8:754–63. doi: 10.1530/EC-19-0045, PMID: 31071680 PMC6547306

[18] Coperchini F De Luca F Greco A Chiardi I Croce L Teliti M. Sexual dimorphism in thyroid cancer: evidence from preclinical studies. Endocr Relat Cancer. (2025) 32:101530. doi: 10.1530/ERC-24-0348, PMID: 40197424

[19] Denaro N Romanò R Alfieri S Dolci A Licitra L Nuzzolese I. The tumor microenvironment and the estrogen loop in thyroid cancer. Cancers. (2023) 15:2458. doi: 10.3390/cancers15092458, PMID: 37173925 PMC10177023

[20] Choi MH Moon JY Cho SH Chung BC Lee EJ. Metabolic alteration of urinary steroids in pre- and post-menopausal women, and men with papillary thyroid carcinoma. BMC Cancer. (2011) 342:1471–2407-11-342. doi: 10.1186/1471-2407-11-342, PMID: 21824401 PMC3199870

[21] Muraoka H Kaneda T Ito K Otsuka K Tokunaga S. Early detection of acute mandibular osteomyelitis using computed tomography texture analysis. Oral Surg Oral Med Oral Pathol Oral Radiol. (2025) 7:40850861. doi: 10.1016/j.oooo.2025.07.008, PMID: 40850861

[22] Chen Z Chen M Huang S Wang Z Zhang Y Huang Y. Texture-based classification of fetal growth restriction from intrauterine neurosonographic image. J Ultras Med. (2024) 44:177–88. doi: 10.1002/jum.16594, PMID: 39365033

[23] Szczypinski PM Strzelecki M Materka A Klepaczko A. MaZda:a soft ware package for image texture analysis. Comput Meth Progr BioMed. (2009) 94:66–76. 10.1016/j.cmpb.2008.08.005 18922598

[24] Ardakani AA Gharbali A Mohammadi A. Classification of benign and Malignant thyroid nodules using wavelet texture analysis of sonograms. J Ultrasound Med. (2015) 34:1983–9. doi: 10.7863/ultra.14.09057, PMID: 26396168

[25] Zhang Q Liu BJ Ren WW He YP Li XL Zhao CK. Association between BRAF V600E mutation and ultrasound features in papillary thyroid carcinoma patients with and without Hashimoto’s thyroiditis. Sci Rep. (2017) 7:4899–9. doi: 10.1038/s41598-017-05153-y, PMID: 28687736 PMC5501791

[26] Campennì A Ruggeri RM Giuffrè G Siracusa M Alibrandi A Cardile D. BRAFV600E mutation is associated with increased prevalence of contralateral lymph-node metastases in low and low-to-intermediate risk papillary thyroid cancer. Nucl Med Commun. (2021) 42:611–8. doi: 10.1097/MNM.0000000000001386, PMID: 33625185

[27] Park VY Lee E Lee HS Kim HJ Yoon J Son J. Combining radiomics with ultrasound-based risk stratification systems for thyroid nodules: an approach for improving performance. Eur Radiol. (2021) 31:2405–13. doi: 10.1007/s00330-020-07365-9, PMID: 33034748

[28] Abbasian AA Gharbali A Mohammadi A. Application of texture analysis method for classification of benign and Malignant thyroid nodules in ultrasound images. Iran J Cancer Prev. (2015) 8:116–24., PMID: 25960851 PMC4411473

[29] Bhatia KS Lam AC Pang SW Wang D Ahuja AT. Feasibility study of texture analysis using ultrasound shear wave elastography to predict Malignancy in thyroid nodules. Ultrasound Med Biol. (2016) 42:1671–80. doi: 10.1016/j.ultrasmedbio.2016.01.013, PMID: 27126245

[30] Zhang H Hu S Wang X He J Liu W Yu C. Prediction of cervical lymph node etastasis using MRI radiomics approach in papillary thyroid carcinoma: a feasibility study. Technol Cancer Res Treat. (2020) 19:1533033820969451. doi: 10.1177/1533033820969451, PMID: 33161833 PMC7658511

[31] Tomita H Kuno H Sekiya K Sakai O Li B. Quantitative assessment of thyroid nodules using Dual-energy computed tomography: iodine concentration measurement and multiparametric texture analysis for differentiating between Malignant and benign lesions. Int J Endocrinol. (2020) 2020:5484671. doi: 10.1155/2020/5484671, PMID: 32256574 PMC7104273

[32] Su GY Xu XQ Zhou Y Zhang H Si Y Shen MP. Texture analysis of dual-phase contrast-enhanced CT in the diagnosis of cervical lymph node metastasis in patients with papillary thyroid cancer. Acta Radiol. (2021) 62:890–6. doi: 10.1177/0284185120946711, PMID: 32757639

[33] Li F Pan D He Y Wu Y Peng J Li J. Using ultrasound features and radiomics analysis to predict lymph node metastasis in patients with thyroid cancer. BMC Surg. (2020) 20:315–21. doi: 10.1186/s12893-020-00974-7, PMID: 33276765 PMC7716434

